# Comparison of Optimization Strategies for Musculoskeletal Modeling of the Wrist for Therapy Planning in Case of Total Wrist Arthroplasty

**DOI:** 10.3390/life12040527

**Published:** 2022-04-02

**Authors:** Jörg Eschweiler, Maximilian Praster, Valentin Quack, Jianzhang Li, Björn Rath, Frank Hildebrand, Filippo Migliorini

**Affiliations:** 1 Department of Orthopaedics, Trauma and Reconstructive Surgery, RWTH Aachen University Hospital, 52074 Aachen, Germany; mpraster@ukaachen.de (M.P.); vquack@ukaachen.de (V.Q.); jli@ukaachen.de (J.L.); fhildebrand@ukaachen.de (F.H.); fmigliorini@ukaachen.de (F.M.); 2 Department of Orthopaedic Surgery, Klinikum Wels-Grieskirchen, 4600 Wels, Austria; bjoern.rath@klinikum-wegr.at; 3 Department of Orthopaedic and Trauma Surgery, Eifelklinik St. Brigida, 52152 Simmerath, Germany

**Keywords:** modeling, total wrist arthroplasty, biomechanics, TWA

## Abstract

The human wrist joint is an elegant mechanism. The wrist allows the positioning and orienting of the hand to the forearm. The computational modeling of the human hand, especially of the wrist joint, can reveal important information about biomechanical mechanisms and provide the basis for its dysfunction and pathologies. For instance, this could be used for therapy planning in total wrist arthroplasty (TWA). In this study, different optimization methods and sensitivity analyses of anatomical parameters for musculoskeletal modeling were presented. Optimization includes finding the best available value of an objective function, including a variety of different types of objective functions. In the simplest case, optimization consists of maximizing or minimizing a function by systematically choosing input values from within an allowed set and computing the value of the function. Optimization techniques are used in many facets, such as the model building of joints or joint systems such as the wrist. The purpose of this study is to show the variability and influence of the included information for modeling, investigating the biomechanical function and load situation of the joint in representative scenarios. These possibilities to take them into account by an optimization and seem essential for the application of computational modeling to joint pathologies.

## 1. Introduction

The wrist is used in many ways and in many different situations in our daily lives; due to that, it is often involved in injuries, diseases, or deformations that may affect the quality of life. In diseases such as rheumatoid arthritis (RA), the wrist joint is frequently painful with impaired motion. Furthermore, it is involved in approximately 25% of all sports-related injuries [1]. It is necessary to objectively quantify parameters that affect wrist function to evaluate functional impairment and the efficacy of therapeutic interventions, such as total wrist arthroplasty (TWA), general wrist surgery, or rehabilitation approaches/programs following acute or chronic wrist ailments.

“Knowledge of the magnitudes and directions of the forces passing through the human joint is essential in the design of many surgical and therapeutic treatment programs” [2]. This missing information is still a current problem and has an interest in musculoskeletal/biomechanical modeling (MBM).

In the case of focusing on the characterization of the wrist joint, one important point is the investigation of the relationship between body motion and biomechanical loads. Here, the intention is to determine optimal posture and exercises during physiological activities (e.g., activities of daily living, ergonomics, training, and also rehabilitation). Additionally, this information could be used to design implants and to prevent, evaluate, and manage wrist joint disorders (e.g., joint deformities because of RA and surgical fixation strategies).

MBM can give insights into the physiological/mechanical behaviour of the joints. Subject-specific biomechanical models are essential for future clinical applications, such as predicting the effects of orthopedic surgery. MBM and simulations may be useful tools to solve complex biomechanical problems, revolutionizing medical decision-making and surgical management strategies. The simulation of the wrist joint is integral to understanding the influence of soft tissue. For example, how muscles behave to move the skeleton in normal and pathological states [3]. Delp et al. stated that a biomechanical description of the wrist’s surrounding muscles is needed to help design, e.g., tendon transfer surgeries [4]. The MBM of the wrist can reveal facts about its biomechanical mechanisms and load application, providing the basis for investigating dysfunction [5].

An open issue in MBM remains to solve the muscular redundancy problem [6]. If the diversity of musculoskeletal function is considered, several different criteria for muscle selection may likely be utilized for different possible activities [7]. All linear or nonlinear optimization procedures assume that the body selects muscles for a given activity according to some criteria (e.g., minimization of muscle force) [7]. The potential of multi-objective optimization, when both musculo-tendon forces and joint reaction forces are minimized, is still unclear. Considering more than one objective function in the optimization problem introduces additional degrees of freedom (DOF) that can stand for the compromise between musculo-tendon and joint reaction forces. The choice of the objective function used to solve the muscular redundancy problem is the bottleneck.

Besides this problem, biomechanical models of the wrist/forearm system may provide an alternative approach to estimating the relative changes in strength and the applied load to the joint [8]. Building a complete biomechanical model of the wrist is very complicated because of its anatomy, including muscles, and the high number of DOF [5]. Sometimes, this complex approach can be very time-consuming.

This study aims to introduce, based on a simple planar model of the wrist joint, the ensemble of optimal solutions and the solution of a global objective method for the following emblematic three-objective problem: optimizing (and minimizing) musculo-tendon forces in a static situation. In this study, we propose possibilities for performing the concurrent muscle problem. We provide different illustrative examples to demonstrate the implementation of the optimization approach to the wrist. Furthermore, different anatomical parameters and their sensitivity in the case of modeling were presented and their possible influence on the results was demonstrated. The aim of the sensitivity analysis was to investigate inaccuracies in muscle modeling parameters and the development of concepts to reduce related error sources.

## 2. Material and Methods

### 2.1. Sensitivity Analysis

#### 2.1.1. General Background of a Sensitivity Analysis

The sensitivity analysis (SA) evaluates the uncertainty in the output of a mathematical model. It can be divided and allocated into different sources of uncertainty in its inputs. However, the changing, e.g., the quality of X-ray images or other (imaging/geometrical) information, and the subjectivity of the included data; furthermore, the subjectivity of the users mandatorily of medical experts lead to inaccuracies in e.g., landmark identification. The extent and effect of these inaccuracies on the MBM output are of interest concerning the robustness of the idealization approach itself and the reliability in the case of the performed therapy planning.

For this approach, MATLAB (R2020a, Mathworks) was used, a well-established numerical computing environment.

#### 2.1.2. Determination of Anatomical and Modeling Information

MBM of musculoskeletal structures requires accurate data on anatomical parameters e.g., anatomical landmarks, muscle lengths (MLs), moment arms (MAs), and those parameters describing the segment position.

The main functions of the wrist are to provide mobility and sustain the load. It is reasonable to assume that mechanical factors play an important role in the function of the wrist joint. To determine these mechanical factors, especially muscle loads, motions, and external loads have to be quantitated in precise mechanical terms. Therefore, the knowledge of anatomical and relevant input parameters is important for our example, which focuses primarily on muscles.

#### 2.1.3. Physiological Cross-Sectional Area

The physiological cross-sectional area (PCSA) of a muscle is a measure of the number of sarcomeres in parallel with the angle of pull of the muscles [9]. In pennate muscles, the fibers act at an angle from the long axis. The angle between the orientation of the muscle fiber and the angle of the muscle’s line of action is called the pennation angle [9]. For each muscle with a parallel-fibered structure, the PCSA can be calculated by the following equation:

(1) $$\mathrm{PCSA}\;\left({\mathrm{mm}}^{2}\right)=\frac{\mathrm{mass}\;\left(g\right)}{\mathrm{density}\;\left(\frac{g}{{\mathrm{mm}}^{3}}\right)\cdot \mathrm{optimum}\;\mathrm{fiber}\;\mathrm{length}\;\left(\mathrm{mm}\right)}$$

In pennate muscles, the PCSA becomes:

(2) $$\mathrm{PCSA}\;\left({\mathrm{mm}}^{2}\right)=\frac{\mathrm{mass}\;\left(g\right)\cdot \cos\theta \left(\mathrm{degree}\right)}{\mathrm{density}\;\left(\frac{g}{{\mathrm{mm}}^{3}}\right)\cdot \mathrm{optimum}\;\mathrm{fiber}\;\mathrm{length}\;\left(\mathrm{mm}\right)}$$

where θ is the pennation angle that increases as the muscle shortens. As in the previous work of Borst et al. [10], a muscle density of 0.0010567 $\frac{g}{{\mathrm{mm}}^{3}}$ was assumed [5]. The PCSA was taken from the literature.

#### 2.1.4. Peak Force Calculation (PFC)

The peak force is calculated as the product of PCSA and the specific tension *σ* as follows:

(3) $$\mathrm{PFC}\;\left(N\right)=\mathrm{PCSA}\left({\mathrm{mm}}^{2}\right)\cdot \sigma \left(\frac{N}{{\mathrm{mm}}^{2}}\right)$$

A wide range of tension values for skeletal muscles has been reported, e.g., [9,11]. Most of these tension values were measured during isometric conditions and ranged from 0.2 to 1.0 $\frac{N}{{\mathrm{mm}}^{2}}$. These higher values were recorded in pennate muscles, which are those whose fibers lie at an angle from the main axis of the muscle [9]. Such an orientation effectively increases the PCSA above that measured and used in the stress calculation [9].

### 2.2. General Approach of MBM

MBM is a noninvasive way to investigate the relationship between body motion and internal biomechanical parameters in a wide range of conditions. Validated models reduce the need for experimental studies, providing useful information about joint kinematics, joint contact pressures/forces, soft tissue tensions, and range of motion (ROM) [12]. Knowledge of muscle and joint contact forces would be highly beneficial from a clinical perspective, but their direct measurement is not feasible in a clinical setting [13]. MBM potentially overcomes the limitations of current practice, and the treatments are based on subjective, static, and mostly qualitative assessments [14].

In the context of the wrist joint, this technique is a valuable approach to examine the effect of changes in muscle force on loads and the risk of wrist pain. Changes in muscle forces have not been properly simulated by previous models. The estimation of general and especially individual muscle forces and their contribution to joint moments is essential for understanding how humans solve the dynamics of movement [15].

**Figure 1:**
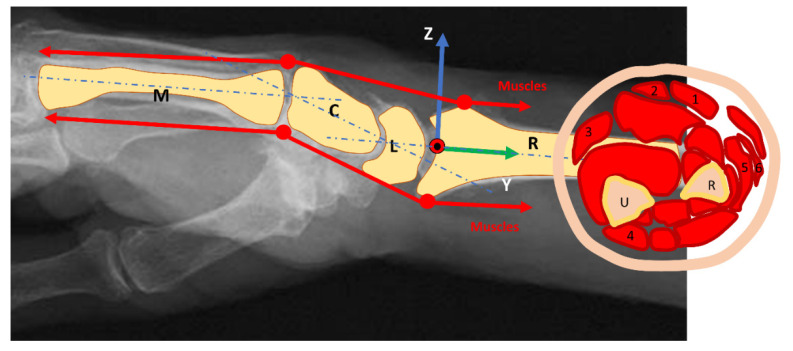
Muscles direction and wrist joint moment arms: The force direction is parallel to the forearm so that the physiological cross-sectional area of the muscle is the leading parameter. (1): M. flexor carpi radialis, (2): M. palmaris longus, (3): M. flexor carpi ulnaris, (4): M. extensor carpi ulnaris, (5–6): Mm. extensors carpi radialis longus et brevis; U: Ulna; R: Radius; M: Metacarpal bone; C: Capitatum; L: Lunatum.

We performed a static study for the (mechanical) system composed of the forearm and wrist, assumed with negligible weight (Figure 1).

The wrist is approximated as a perfect hinge joint for flexion and extension and radial and ulnar deviation according to the state of the art [16]. Six muscles realize the following main motion: M. flexor carpi radialis (FCR), M. palmaris longus (PL), M. flexor carpi ulnaris (FCU), M. extensor carpi ulnaris (ECU), Mm. extensors carpi radialis longus et brevis (ECRL and ECRB) [16,17,18,19,20]. The flexion is mainly produced by the PL, FCU, and FCR. The motion is supported by the flexor digitorum superficialis muscle. The extension is mainly produced by the ECRL, ECRB, and ECU. They will be assisted from the extensor digitorum muscle. The abduction muscles of the wrist (radial deviation) are the FCR, the ECRL, and the ECRB, and the abductor pollicis longus supports the motion. Muscles involved in the adduction of the wrist (ulnar deviation) are the FCU and the ECU.

**Figure 2:**
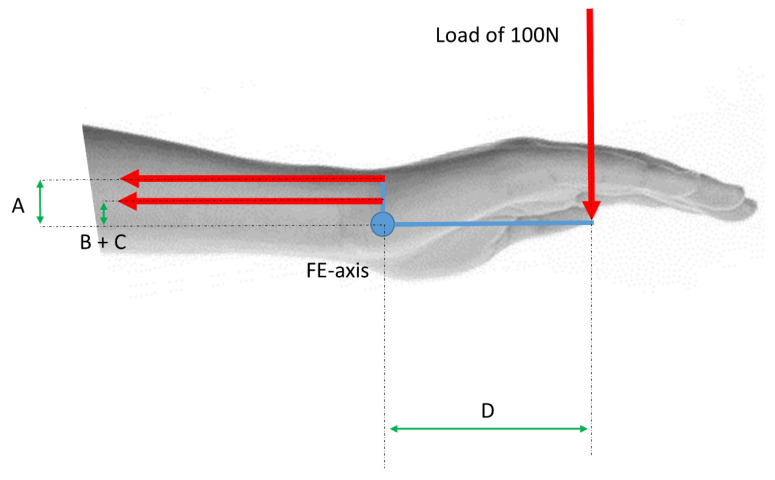
Free body diagram: (A): lever arm of ECRB; (B–C): lever arm of ECU and ECRL; (D): lever arm (10 cm = 0.1 m) of an applied load of 100 N.

For an exemplar computation, a maximum load of 100 N was applied to the wrist with a lever arm of 10 cm (=0.1 m), which resulted in a moment of 10 N·m (=1000 N·cm) (Figure 2). To hold the joint in static equilibrium in a linear case, the forces for each muscle must be calculated e.g., for flexion. That means, to ensure an equilibrium the extensor muscles ECU, ECRB, and ECRL must hold the hand with 10 Nm.

What makes this system indeterminate is the fact that there exist more unknowns than solvable equations. This means the system is (statically) indeterminate. There are many possible combinations of values for the muscle forces that will satisfy the equilibrium equation. The study and treatment of these conditions could greatly benefit from combined software tools that offer better insights into neuromuscular biomechanics, and predictive capabilities for optimal surgical treatment planning [21].

An open issue in MBM is solving the muscular redundancy problem [6]. This problem states that there are multiple ways to perform a movement with different muscle activations. In other words, having more muscles than DOFs [22]. Under normal circumstances, no simple one-to-one correspondence exists between a muscular redundancy problem and a solution to the problem.

There are the following two widely used approaches to determining a unique solution: (1) reduction methods and (2) optimization methods. The reduction methods try to reduce the number of unknowns via the introduction of additional boundary constraints to obtain an equal number of equations. For example, if two muscles have the same attachment and perform a similar task, they could be seen as one muscle. This reduces the number of unknown muscle forces/parameters by one. The attractiveness of the reduction method is that the same technique used to solve the muscle force problem could be used.

#### 2.2.1. Calculating Static Muscle Forces Using a General Optimization Approach Based on the Weighted Sum Method

An alternative to the reduction methods are optimization methods. One common approach to the force distribution problem is to formulate a static optimization model.

First, a weighted sum method is proposed. In this approach the following percentual part of the muscles for the flexion motion of the wrist in proportion to the main muscle cross-sectional was considered for the calculation:

(4) $$M=\sum_{i=1}^{m}\left(d_{i}\cdot \frac{A_{i}}{A}F\right)$$

(5) F≥0

(6) $$A=\sum_{i=1}^{m}A_{i}=1$$

where F denotes the unknown muscle forces of ith forearm muscle, A_i_ denotes the cross-sectional area of the ith muscle, M the wrist joint moment, d_i_ the moment arm of the ith muscle of the wrist, m is the number of muscles involved in the motion (m = 3), and A is the main/the summation cross-sectional area of all involved muscles (A = 1 = 100%).

#### 2.2.2. Calculating Static Muscle Forces Using Optimization Based on an Approach by Crowninshield and Brand

The approach proposed by Crowninshield and Brand is surely the most used optimization model in the literature [7]. Here, the cost function takes the form of a weighted sum of muscle forces raised to some non-linear power. The weighting is based on constant values for the PCSA. The basis is that every physical system reaches its equilibrium state by the energy minimization principle. Transferred to the body this means that the muscles are activated in such a way that fatigue is minimized.

The principle is to minimize the so-called objective function U [7]:

(7) Minimize {U}

The objective function U proposed by Crowninshield and Brand is given as follows [7]:

(8) $$U=\sum_{i=1}^{m}{\left(\frac{F_{i}}{{\mathrm{PCSA}}_{i}}\right)}^{n}$$

subject to:

(9) $$M-\sum_{i=1}^{m}\left(d_{i}\cdot F_{i}\right)=0\;\to M=\sum_{i=1}^{m}\left(d_{i}\cdot F_{i}\right)\;F_{i}\geq 0\;i=1\;\mathrm{to}\;m$$

where F_i_ denotes the unknown muscle forces of ith forearm muscle, PCSA_i_ denotes the physiological cross-sectional area of the ith muscle, M the wrist joint moment, d_i_ the moment arm vector of the ith muscle at the wrist, m is the number of muscles crossing the joint, and *n* is the exponent in the muscle endurance-force relationship. This norm is minimized when the radicand (summation of the nth power of muscle stresses) is minimized.

Depending on the value of n, the Crowninshield and Brand criteria may yield a linear programming problem (*n* = 1), a quadratic programming problem (*n* = 2), or a general nonlinear programming problem (*n* > 2).

Considering the optimization formulation applied to the indeterminate problem of an applied force of 100 N in case of flexion to the hand and the distance to the rotational center is D = 10 cm (Figure 2). For this approach, MATLAB (R2020a, Mathworks) was used. It includes hundreds of general-purpose and specialized functions for numerical computation, organized into libraries called toolboxes that cover such areas as simulation, optimization, systems, and control and signal processing. If Equation (9) is taken and insert the resulting moment, it follows:

(10) $$1000-\sum_{i=1}^{m}\left(d_{i}\cdot F_{i}\right)=0\;\to 1000=\sum_{i=1}^{m}\left(d_{i}\cdot F_{i}\right)\;F_{i}\geq 0\;i=1\;\mathrm{to}\;3$$

In the case of following Crowninshield and Brand and assuming *n* = 1 and m = 3, the following optimization problem will be originated [7]:

(11) $$\begin{array}{c} \mathrm{Min}\\ \left(F_{\mathrm{ECU}}{\;F}_{\mathrm{ECRB}}{\;F}_{\mathrm{ECRL}}\right) \end{array}=\sum_{i=1}^{3}{\left(\frac{F_{i}}{{\mathrm{PCSA}}_{i}}\right)}^{1}=\left(\frac{F_{\mathrm{ECU}}}{{\mathrm{PCSA}}_{\mathrm{ECU}}}\right)+\left(\frac{F_{\mathrm{ECRB}}}{{\mathrm{PCSA}}_{\mathrm{ECRB}}}\right)+\left(\frac{F_{\mathrm{ECRL}}}{{\mathrm{PCSA}}_{\mathrm{ECRL}}}\right)$$

subject to:

(12) $$M=\sum_{i=1}^{3}\left(d_{i}\cdot F_{i}\right)=\left(d_{\mathrm{ECU}}\cdot F_{\mathrm{ECU}}\right)+\left(d_{\mathrm{ECRB}}\cdot F_{\mathrm{ECRB}}\right)+\left(d_{\mathrm{ECRL}}\cdot F_{\mathrm{ECRL}}\right)$$

In addition,

(13) $$\{\begin{array}{c} A_{\mathrm{eq}}=b_{\mathrm{eq}}\\ A_{\mathrm{ineq}}\geq 0 \end{array}$$

The problem can be classified as a linear problem. The objective function and the constraints are linear functions of the minimizing variables F_ECU_, F_ECRB_, and F_ECRL_. To utilize MATLAB for solving the linear programming problem, the following “linprog” solver was used:

(14) $${\left[\begin{array}{ccc} \frac{1}{{\mathrm{PCSA}}_{\mathrm{ECU}}} & \frac{1}{{\mathrm{PCSA}}_{\mathrm{ECRB}}} & \frac{1}{{\mathrm{PCSA}}_{\mathrm{ECRL}}} \end{array}\right]}^{T}\left[\begin{array}{c} F_{\mathrm{ECU}}\\ F_{\mathrm{ECRB}}\\ F_{\mathrm{ECRL}} \end{array}\right]$$

subject to:

(15) $$\left[\begin{array}{ccc} d_{\mathrm{ECU}} & d_{\mathrm{ECRB}} & d_{\mathrm{ECRL}} \end{array}\right]\left[\begin{array}{c} F_{\mathrm{ECU}}\\ F_{\mathrm{ECRB}}\\ F_{\mathrm{ECRL}} \end{array}\right]=M=1000\;N\cdot \mathrm{cm}$$

where:

$$A_{\mathrm{eq}}=\left[\begin{array}{ccc} d_{\mathrm{ECU}} & d_{\mathrm{ECRB}} & d_{\mathrm{ECRL}} \end{array}\right]\;x=\left[\begin{array}{c} F_{\mathrm{ECU}}\\ F_{\mathrm{ECRB}}\\ F_{\mathrm{ECRL}} \end{array}\right]\;b_{\mathrm{eq}}=M$$

If we assume *n* = 2 and m = 3, the following optimization problem will be originated:

(16) $$\begin{array}{c} \mathrm{Min}\\ \left(F_{\mathrm{ECU}}{\;F}_{\mathrm{ECRB}}{\;F}_{\mathrm{ECRL}}\right) \end{array}=\sum_{i=1}^{3}{\left(\frac{F_{i}}{{\mathrm{PCSA}}_{i}}\right)}^{2}={\left(\frac{F_{\mathrm{ECU}}}{{\mathrm{PCSA}}_{\mathrm{ECU}}}\right)}^{2}+{\left(\frac{F_{\mathrm{ECRB}}}{{\mathrm{PCSA}}_{\mathrm{ECRB}}}\right)}^{2}+{\left(\frac{F_{\mathrm{ECRL}}}{{\mathrm{PCSA}}_{\mathrm{ECRL}}}\right)}^{2}$$

subject to:

(17) $$M=\sum_{i=1}^{3}\left(d_{i}\cdot F_{i}\right)=\left(d_{\mathrm{ECU}}\cdot F_{\mathrm{ECU}}\right)+\left(d_{\mathrm{ECRB}}\cdot F_{\mathrm{ECRB}}\right)+\left(d_{\mathrm{ECRL}}\cdot F_{\mathrm{ECRL}}\right)$$

For the case of a quadratic approach it follows:

(18) $$\frac{1}{2}{\left[F_{\mathrm{ECU}}{\;F}_{\mathrm{ECRB}}{\;F}_{\mathrm{ECRL}}\right]}^{T}\left[\begin{array}{ccc} \frac{2}{{\mathrm{PCSA}}_{\mathrm{ECU}}{}^{2}} & 0 & 0\\ 0 & \frac{2}{{\mathrm{PCSA}}_{\mathrm{ECRB}}{}^{2}} & 0\\ 0 & 0 & \frac{2}{{\mathrm{PCSA}}_{\mathrm{ECRL}}{}^{2}} \end{array}\right]\left[\begin{array}{c} F_{\mathrm{ECU}}\\ F_{\mathrm{ECRB}}\\ F_{\mathrm{ECRL}} \end{array}\right]$$

where:

$$x^{T}=\frac{1}{2}{\left[F_{\mathrm{ECU}}{\;F}_{\mathrm{ECRB}}{\;F}_{\mathrm{ECRL}}\right]}^{T}\;H=\left[\begin{array}{ccc} \frac{2}{{\mathrm{PCSA}}_{\mathrm{ECU}}{}^{2}} & 0 & 0\\ 0 & \frac{2}{{\mathrm{PCSA}}_{\mathrm{ECRB}}{}^{2}} & 0\\ 0 & 0 & \frac{2}{{\mathrm{PCSA}}_{\mathrm{ECRL}}{}^{2}} \end{array}\right]\;x=\left[\begin{array}{c} F_{\mathrm{ECU}}\\ F_{\mathrm{ECRB}}\\ F_{\mathrm{ECRL}} \end{array}\right]$$

subject to:

(19) $$\left[\begin{array}{ccc} d_{\mathrm{ECU}} & d_{\mathrm{ECRB}} & d_{\mathrm{ECRL}} \end{array}\right]\left[\begin{array}{c} F_{\mathrm{ECU}}\\ F_{\mathrm{ECRB}}\\ F_{\mathrm{ECRL}} \end{array}\right]=1000\;N\cdot \mathrm{cm}$$

In the case where *n* > 2, e.g., if *n* = 3, the optimization problem may be stated as follows:

(20) $$\begin{array}{c} \mathrm{Min}\\ \left(F_{\mathrm{ECU}}{\;F}_{\mathrm{ECRB}}{\;F}_{\mathrm{ECRL}}\right) \end{array}=\sum_{i=1}^{3}{\left(\frac{F_{i}}{{\mathrm{PCSA}}_{i}}\right)}^{3}={\left(\frac{F_{\mathrm{ECU}}}{{\mathrm{PCSA}}_{\mathrm{ECU}}}\right)}^{3}+{\left(\frac{F_{\mathrm{ECRB}}}{{\mathrm{PCSA}}_{\mathrm{ECRB}}}\right)}^{3}+{\left(\frac{F_{\mathrm{ECRL}}}{{\mathrm{PCSA}}_{\mathrm{ECRL}}}\right)}^{3}$$

subject to the following:

(21) $$M=\sum_{i=1}^{3}\left(d_{i}\cdot F_{i}\right)=\left(d_{\mathrm{ECU}}\cdot {\;F}_{\mathrm{ECU}}\right)+\left(d_{\mathrm{ECRB}}\cdot {\;F}_{\mathrm{ECRB}}\right)+\left(d_{\mathrm{ECRL}}\cdot {\;F}_{\mathrm{ECRL}}\right)$$

Now it is a general nonlinear programming problem. The reason for that is the nonlinearity of the objective function. To solve this problem, the “fmincon” solver in MATLAB was used.

#### 2.2.3. Calculating Static Muscle Forces Using an Individual Optimization Approach

An alternative optimization approach was published by An et al. [2]. Here, a linear approach was presented with additional constraints that only a few muscles will be active during a special movement.

(22) Min σ

Subject to the following:

(23) $$\frac{F_{i}}{{\mathrm{PCSA}}_{i}}\leq \sigma$$

in which PCSA represents the physiological cross-sectional area of the ith muscle, and the left-hand side of the equation represented the “stress” (σ_i_) of the ith muscle.

The resultant joint forces and moments were then obtained by the remaining force and moment equilibrium equations. The flexion-extension moment equations are as follows:

(24) $$\sum d_{i}\cdot F_{i}=M$$

in which M represented the intersegmental moments due to externally applied load in flexion-extension about the joint center. The moment arms in flexion-extension of the ith muscle were d_i_. F_i_ represents the magnitude of the ith unknown muscle force.

An et al. justified their method based on the fact that nonlinear programming methods can be very sensitive to ill-conditioned data [2]. In MATLAB, this type of problem will be solved using the “fminimax” solver.

## 3. Results

### 3.1. Determination of Anatomical Parameters for Analyzing the Wrist Joint

#### 3.1.1. Anatomical Parameters

The different anatomical parameters are shown in Table 1, which are used for analyzing the muscles strength and loads of the wrist joint.

#### 3.1.2. Moment Arms of the Muscles

**Figure 3:**
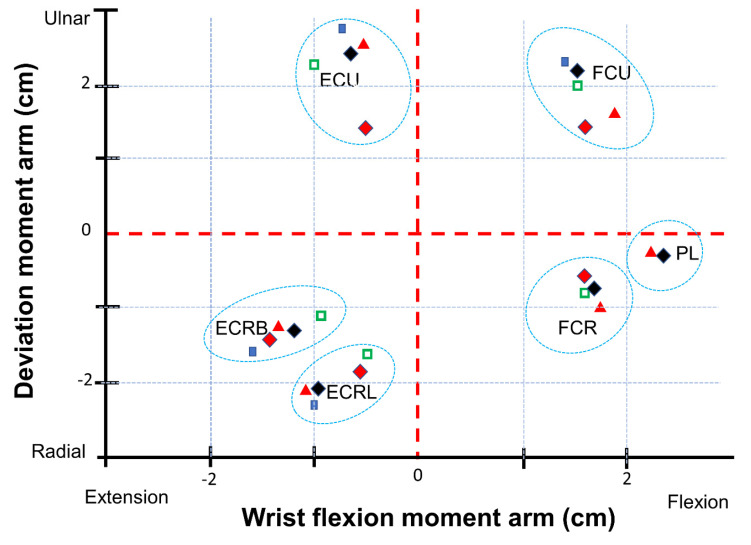
Muscle moment arms: information taken from [11] are shown as black diamonds, the parameters shown as blue quadrat were taken from [28], the parameters shown as green quadrat were taken from [29], the parameters shown as red triangle were taken from [30], the parameters shown as red diamonds were taken from [31]. (M. flexor carpi radialis (FCR), M. palmaris longus (PL), M. flexor carpi ulnaris (FCU), M. extensor carpi ulnaris (ECU), Mm. extensors carpi radialis longus et brevis (ECRL and ECRB)).

Figure 3 shows the technical information for the different muscles during wrist motion.

### 3.2. Sensitivity Analysis

Table 2 shows the parameters for the sensitivity analysis.

**Figure 4:**
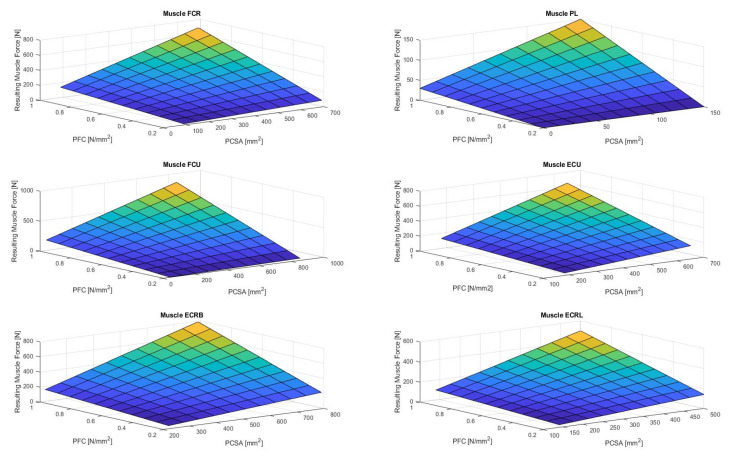
Resulting wrist muscle force in case of varied different modeling parameters. (M. flexor carpi radialis (FCR), M. palmaris longus (PL), M. flexor carpi ulnaris (FCU), M. extensor carpi ulnaris (ECU), Mm. extensors carpi radialis longus et brevis (ECRL and ECRB); PCSA: physiological cross-sectional area; PFC: Peak force calculation).

**Figure 5:**
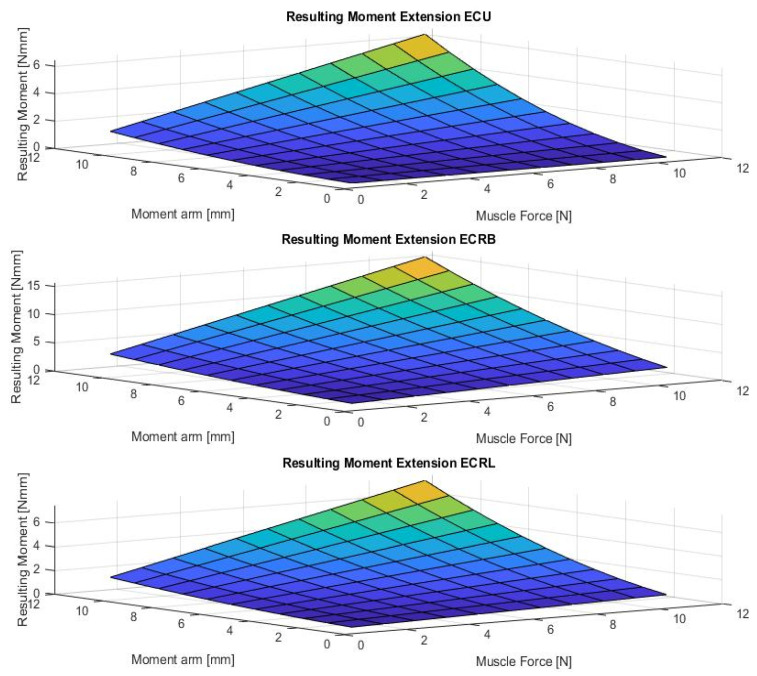
The figure shows the resulting wrist moment during extension in case for different parameters (muscle forces and moment arm). (M. extensor carpi ulnaris (ECU), Mm. extensors carpi radialis longus et brevis (ECRL and ECRB)).

**Figure 6:**
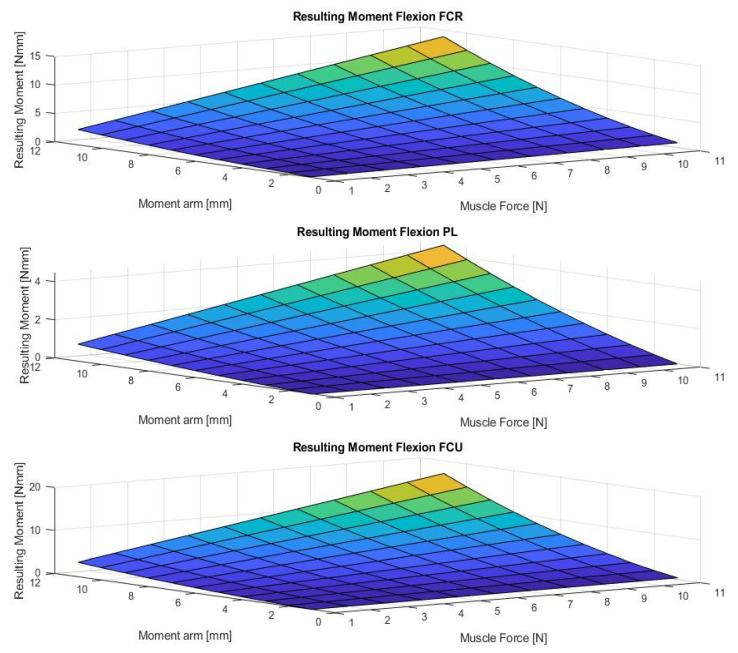
The figure shows the resulting wrist moment during flexion for varying different parameters (muscle forces and moment arms). (M. flexor carpi radialis (FCR), M. palmaris longus (PL), M. flexor carpi ulnaris (FCU)).

**Figure 7:**
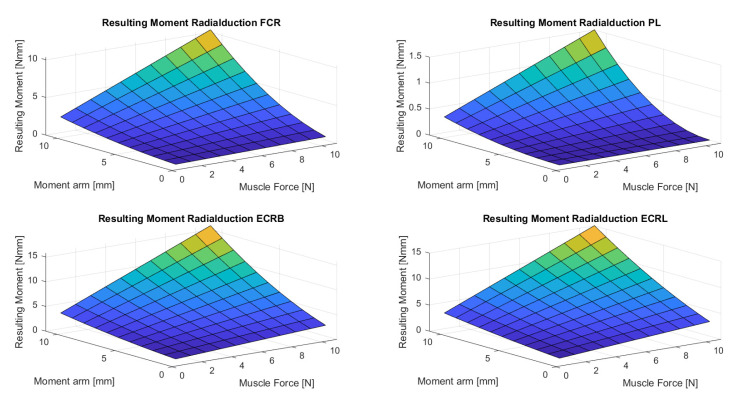
The figure shows the resulting wrist moment during radial deviation for different parameters. The adduction is produced by the ECU and FCU. The abduction is produced by the FCR, ECRL, and ECRB. (M. flexor carpi radialis (FCR), M. palmaris longus (PL), Mm. extensors carpi radialis longus et brevis (ECRL and ECRB)).

**Figure 8:**
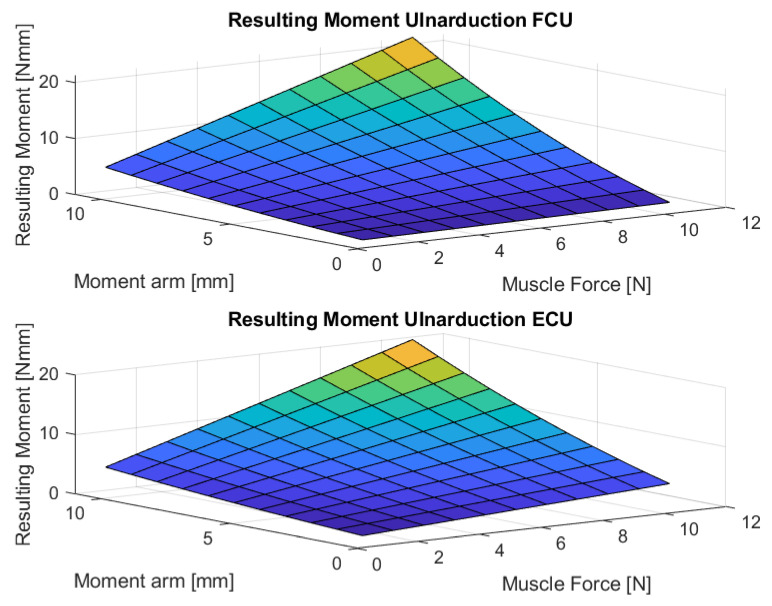
The figure shows the resulting wrist moment during ulnar deviation for different parameters. (M. flexor carpi ulnaris (FCU), M. extensor carpi ulnaris (ECU)).

Figure 4, Figure 5, Figure 6, Figure 7 and Figure 8 show the results of the sensitivity analysis of the wrist joint.

### 3.3. Optimization of the Muscle Redundancy Problem

#### 3.3.1. Results for Using a General Optimization Approach Based on the Weighted Sum Method

If we know to take the mean lever arms for each muscle, e.g., from [31], we will obtain for ECU a lever arm of 6 mm (0.006 m), for ECRB a lever arm of 12 mm (0.012 m), and for ECRL a lever arm of 7 mm (0.007 m). To calculate the part of the force for each muscle, we can choose the PCSA and calculate the percentual part of the summary of the complete cross-sectional area.

For example, if we take the PCSA from [23], we find an area for the ECU of 480 mm^2^ (=4.8 cm^2^), for an ECRB of 450 mm^2^ (=4.5 cm^2^), and for ECRL of 370 mm^2^ (=3.7 cm^2^). The sum of all is then 1300 mm^2^ (=13 cm^2^ = 100%). That means the percentual part for the ECU is 36.9%, for the ECRB it is 34.6%, and for the ECRL it is 28.5%.

We obtained the following:

1000 N·cm=0.6 cm·ECU+1.2 cm·ECRB+0.7 cm·ECRL

With the substitution we obtain the following:

1000 N·cm=0.6 cm·0.37 F+1.2 cm·0.35 F+0.7 cm·0.28 F

That means the following:

1000 N·cm=0.6 cm·0.37 F+1.2 cm·0.35 F+0.7 cm·0.28 F1000 N·cm=0.22 cm·F+0.42 cm·F+0.20 cm·F1000 N·cm=0.84 cm·F1190.48 N=F

That means we have to ensure a muscle force in the sum of 1190.48 N, and for each muscle and a force of the following:

ECU = 440.48 N (=0.37 F)

ECRB = 416.67 N (=0.35 F)

ECRL = 333.33 N (=0.28 F)

#### 3.3.2. Results for Calculating Static Muscle Forces Using Optimization Based on an Approach by Crowninshield and Brand

As these solutions are optimal in the sense that they minimize the utility function *U* as follows: 

For n = 1:

ECU = 0 N

ECRB = 833.34 N

ECRL = 0 N

For n = 2:

ECU = 313.03 N

ECRB = 550.24 N

ECRL = 216.99 N

For n = 3:

ECU = 377.05 N

ECRB = 484.03 N

ECRL = 275.62 N

This example illustrates a concept in biomechanics that muscles generally must generate large forces to balance external loads because of their often very short moment arms. Crowninshield and Brand discussed how, in the case the exponent in the objective function increases, the resulting force is more distributed between the movement-involved muscles to reduce the onset of muscle fatigue [7].

#### 3.3.3. Calculating Static Muscle Forces Using Optimization Based on an Approach by An et al.

As these solutions are optimal in the sense that they minimize the utility function U:

ECU = 441.58 N

ECRB = 413.98 N

ECRL = 340.39 N

## 4. Discussion

The carpus is a challenging and complex mechanical system because of its organization of bones and soft tissue structures. The aim of this in silico investigation is to put forward the idea of multi-objective optimization in MBM and the understanding of the wrist by the recognition that there is no standard carpal mechanical system and no typical wrist. This study investigated the effect of variations in muscle forces acting on the wrist using a simple MBM approach.

### 4.1. Sensitivity Analysis

Indeed, the method proposed in this paper allows comparative statements and is based on information taken out of the literature (Figure 4, Figure 5, Figure 6, Figure 7 and Figure 8). However, differences in the impact of inaccuracies in the modeling parameters on the simulation results could be detected and should be further investigated. They showed a huge variation and could influence in the case of choosing them in a model environment. The extent of scattering observed verified the necessity to perform a sensitivity analysis of the available anatomical information. The strong scattering originates from the individuals who were investigated in the different references, e.g., because of the stress values, which range from 0.2 to 1.0 $\frac{N}{{\mathrm{mm}}^{2}}$.

### 4.2. Optimization

We tried to introduce the different optimization approaches and the idea of MBM via an easily simplified model of the wrist because it is easier to analyze and interpret the effects of different optimizations on a simple model before moving to complex ones [6]. In this case, we are interested in examining the muscle forces using optimization methods; we have to consider some assumptions. The main one is that the solution to the problem is unique to each individual task. The more realistic the approximation is based on physiological or functional parameters, the better the estimation of realistic results [32].

The outcomes indicate that muscle forces had a marked impact on computed wrist loads. The knowledge of the forces passing through the wrist joint is essential in the design of many surgical and therapeutic treatments because normal carpal mechanics relies on the interactions between the muscles, ligaments, and carpal bone morphology. The novel feature of this work is the introduction of different optimization approaches that efficiently emulate the behavior of static load analyses within the optimization’s constraints. The example applications yielded reasonable load predictions. To obtain these results, we had to adjust the optimization constraints. The optimization function is one of the most important points in the optimization process because its minimization or maximization determines the solution to the problem [32]. The objective function must be related to the physical problem, e.g., the physiological and pathological situations, respectively.

We also made sure that the different optimization approaches were able to match the (experimentally/literature-based) obtained loads.

For example, when it was planned to include such an approach in the daily clinical process, the fmincon algorithm from the MATLAB Optimization Toolbox was simple to implement and very fast to be of much practical value. If a computer cluster is available, e.g., fmincon could be run in parallel using the MATLAB Parallel Computing Toolbox [33]. That implies that such an approach produces additional information without elongating the daily clinical workflow.

Contact surfaces and ligaments are not explicitly considered in this investigation. The interactions between the muscular and joint structures, and the possibility to take them into account in MBM, seem essential for applications to joint pathologies [6].

The next step should be that such multi-objective optimization approaches should be evaluated on more complex musculoskeletal models and, in the best case, validated by the results of an instrumented prosthesis. No data for the wrist joint in the context of the instrumented prosthesis have been reported so far in the literature.

### 4.3. Limitation

The replication of wrist motions in vitro using only six tendons was a limitation of this study. These tendons were selected since they insert at the base of the metacarpals, thereby primarily affecting the wrist. All muscles contributing to wrist torque, including the extrinsic muscles of the fingers and thumb, should be considered for future MBM approaches. The inclusion of one type of objective function, one joint without an angle and in the neutral position, respectively, and one external moment does not fully represent reality.

Because of the consideration of different optimization criteria, a comparison of the criteria and the resulting outcome is difficult because of the following: (1) the possible different formulation of the problem; (2) the different model assumptions used; (3) the different accuracy and reliability of the input data (e.g., PCSA, error in measurement data); (4) the different optimization algorithms used; (5) the use of different degrees of freedom according to the complexity of the model [32].

Furthermore, the planar model of the wrist used in this study seemed appropriate for an exploratory study of multi-objective optimization but is not appropriate for validation purposes. Therefore, future work should preferentially consist of multi-objective optimizations applied to more complex models of the wrist.

## 5. Conclusions

A comprehensive, unifying model of the functional kinematics of the carpus remains elusive. The complexity involved both in determining the input parameters and the interaction between the muscles involved is non-trivial.

The MATLAB options provide a powerful and flexible approach for generating optimal simulations of musculoskeletal parameters using the implemented optimization approaches. This should facilitate the use of these methods in developing therapies and devices for clinical conditions. The approach presented here shows that numerical simulations play an important role in solving complex engineering problems. Moreover, they have the potential to revolutionize medical decision-making and treatment strategies.

## Figures and Tables

**Table 1:** The presented information was taken out of the literature. There exists a huge variation of the detected data.

Muscle	Volume	SD	Fiber Length	SD	Tendon Length	SD	Pennation Angle	SD	Mass	SD	PCSA	SD	Reference	*n*
	[cm^3^]		[mm]		[mm]		[deg]		[g]		[mm^2^]			
FCR	0.00	0.00	**76.40**	0.00	**188.00**	0.00	0.00	0.00	**7.40**	0.00	**91.60**	0.00	[5]	**1**
	**12.40**	**4.90**	**58.00**	9.00	0.00	0.00	0.00	0.00	0.00	0.00	**200.00**	**60.00**	[23]	**6**
	0.00	0.00	**52.00**	0.00	0.00	0.00	0.00	0.00	0.00	0.00	0.00	0.00	[24]	**15**
	0.00	0.00	**49.00**	0.00	0.00	0.00	0.00	0.00	**35.00**	0.00	**681.00**	0.00	[25]	**1**
	0.00	0.00	**63.00**	0.00	**244.00**	0.00	**3.00**	0.00	0.00	0.00	**160.00**	0.00	[11]	*
	0.00	0.00	**164.00**	0.00	0.00	0.00	**3.10**	0.00	0.00	0.00	**199.00**	0.00	[26]	*
	0.00	0.00	0.00	0.00	0.00	0.00	0.00	0.00	0.00	0.00	0.00	0.00	[3]	**1**
	0.00	0.00	0.00	0.00	0.00	0.00	0.00	0.00	0.00	0.00	**199.00**	0.00	[27]	**18**
PL	0.00	0.00	**89.20**	0.00	**247.00**	0.00	0.00	0.00	**3.40**	0.00	**36.10**	0.00	[5]	**1**
	**5.10**	**3.90**	**57.00**	**10.00**	0.00	0.00	0.00	0.00	0.00	0.00	**90.00**	**60.00**	[23]	**6**
	0.00	0.00	**50.00**	0.00	0.00	0.00	0.00	0.00	0.00	0.00	0.00	0.00	[24]	**15**
	0.00	0.00	0.00	0.00	0.00	0.00	0.00	0.00	0.00	0.00	0.00	0.00	[25]	**1**
	0.00	0.00	**64.00**	0.00	**269.00**	0.00	**4.00**	0.00	0.00	0.00	**60.00**	0.00	[11]	*
	0.00	0.00	**134.00**	0.00	0.00	0.00	**3.50**	0.00	0.00	0.00	**69.00**	0.00	[26]	*
	0.00	0.00	0.00	0.00	0.00	0.00	0.00	0.00	0.00	0.00	0.00	0.00	[3]	**1**
	0.00	0.00	0.00	0.00	0.00	0.00	0.00	0.00	0.00	0.00	**69.00**	0.00	[27]	**18**
FCU	0.00	0.00	**72.80**	0.00	**200.00**	0.00	**10.00**	0.00	**13.30**	0.00	**173.00**	0.00	[5]	**1**
	**15.20**	0.00	**48.00**	0.00	0.00	0.00	0.00	0.00	0.00	0.00	**47.00**	0.00	[23]	**6**
	0.00	0.00	**42.00**	0.00	0.00	0.00	0.00	0.00	0.00	0.00	0.00	0.00	[24]	**15**
	0.00	0.00	**56.00**	0.00	0.00	0.00	0.00	0.00	**49.70**	0.00	**842.00**	0.00	[25]	**1**
	0.00	0.00	**51.00**	0.00	**265.00**	0.00	**12.00**	0.00	0.00	0.00	**290.00**	0.00	[11]	*
	0.00	0.00	**228.00**	0.00	0.00	0.00	**12.10**	0.00	0.00	0.00	**342.00**	0.00	[26]	*
	**26.80**	0.00	**40.30**	**14.50**	0.00	0.00	**15.50**	**7.00**	0.00	0.00	**631.90**	0.00	[3]	**1**
	0.00	0.00	0.00	0.00	0.00	0.00	0.00	0.00	0.00	0.00	0.00	0.00	[27]	**18**
ECU	0.00	0.00	**45.60**	0.00	**137.00**	0.00	**11.00**	0.00	**8.90**	0.00	**184.80**	0.00	[5]	**1**
	**14.90**	**5.70**	**45.00**	**5.00**	0.00	0.00	0.00	0.00	0.00	0.00	**480.00**	**140.00**	[23]	**6**
	0.00	0.00	**45.00**	0.00	0.00	0.00	0.00	0.00	0.00	0.00	0.00	0.00	[24]	**15**
	0.00	0.00	**56.00**	0.00	0.00	0.00	0.00	0.00	**38.20**	0.00	**644.00**	0.00	[25]	**1**
	0.00	0.00	**62.00**	0.00	**228.00**	0.00	**4.00**	0.00	0.00	0.00	**210.00**	0.00	[11]	*
	0.00	0.00	**182.00**	0.00	0.00	0.00	**3.50**	0.00	0.00	0.00	**260.00**	0.00	[26]	*
	0.00	0.00	0.00	0.00	0.00	0.00	0.00	0.00	0.00	0.00	0.00	0.00	[3]	**1**
	0.00	0.00	0.00	0.00	0.00	0.00	0.00	0.00	0.00	0.00	**260.00**	0.00	[27]	**18**
ECRB	0.00	0.00	**35.10**	0.00	**185.00**	0.00	**12.00**	0.00	**9.60**	0.00	**258.80**	0.00	[5]	**1**
	**15.80**	**8.10**	**53.00**	**6.00**	0.00	0.00	0.00	0.00	0.00	0.00	**450.00**	**140.00**	[23]	**6**
	0.00	0.00	**61.00**	0.00	0.00	0.00	0.00	0.00	0.00	0.00	0.00	0.00	[24]	**15**
	0.00	0.00	**49.00**	0.00	0.00	0.00	0.00	0.00	**40.50**	0.00	**781.00**	0.00	[25]	**1**
	0.00	0.00	**59.00**	0.00	**222.00**	0.00	**9.00**	0.00	0.00	0.00	**220.00**	0.00	[11]	*
	0.00	0.00	**127.00**	0.00	0.00	0.00	**8.90**	0.00	0.00	0.00	**273.00**	0.00	[26]	*
	0.00	0.00	0.00	0.00	0.00	0.00	0.00	0.00	0.00	0.00	0.00	0.00	[3]	**1**
	0.00	0.00	0.00	0.00	0.00	0.00	0.00	0.00	0.00	0.00	**273.00**	0.00	[27]	**18**
ECRL	0.00	0.00	**72.70**	0.00	**210.00**	0.00	0.00	0.00	**13.90**	0.00	**171.10**	0.00	[5]	**1**
	**18.30**	**7.50**	**78.00**	**5.00**	0.00	0.00	0.00	0.00	0.00	0.00	**370.00**	**90.00**	[23]	**6**
	0.00	0.00	**93.00**	0.00	0.00	0.00	0.00	0.00	0.00	0.00	0.00	0.00	[24]	**15**
	0.00	0.00	**90.00**	0.00	0.00	0.00	0.00	0.00	**47.40**	0.00	**500.00**	0.00	[25]	**1**
	0.00	0.00	**81.00**	0.00	**224.00**	0.00	0.00	0.00	0.00	0.00	**220.00**	0.00	[11]	*
	0.00	0.00	**93.70**	0.00	0.00	0.00	**2.50**	0.00	0.00	0.00	**146.00**	0.00	[26]	*
	0.00	0.00	0.00	0.00	0.00	0.00	0.00	0.00	0.00	0.00	0.00	0.00	[3]	**1**
	0.00	0.00	0.00	0.00	0.00	0.00	0.00	0.00	0.00	0.00	0.00	0.00	[27]	**18**

* Simulation study: M. flexor carpi radialis (FCR), M. palmaris longus (PL), M. flexor carpi ulnaris (FCU), M. extensor carpi ulnaris (ECU), Mm. extensors carpi radialis longus et brevis (ECRL and ECRB); PCSA: physiological cross-sectional area; SD: Standard deviation; n: numbers of the included specimen.

**Table 2:** Parameters for the SA. (M. flexor carpi radialis (FCR), M. palmaris longus (PL), M. flexor carpi ulnaris (FCU), M. extensor carpi ulnaris (ECU), Mm. extensors carpi radialis longus et brevis (ECRL and ECRB); PCSA: physiological cross-sectional area; PFC: Peak force calculation).

Muscle	PCSA		Moment Arm								PFC	
	[mm^2^]		[mm]								[N/mm^2^]	
			Flex		Ext		RD		UD			
	Min	Max	Min	Max	Min	Max	Min	Max	Min	Max	Min	Max
**FCR**	90	690	10	20	./.	./.	5	15	./.	./.	0.2	1.0
**PL**	30	150	20	30	./.	./.	0	10	./.	./.		
**FCU**	40	850	10	20	./.	./.	./.	./.	15	25		
**ECU**	180	650	./.	./.	0	10	./.	./.	20	30		
**ECRB**	220	790	./.	./.	10	20	10	20	./.	./.		
**ECRL**	140	500	./.	./.	5	15	20	30	./.	./.		
